# Supplementary material for: Influence of Metal Salts Addition on Physical and Electrochemical Properties of Ethyl and Propylammonium Nitrate
Source: Int J Mol Sci. 2022 Dec 16;23(24):16040. doi: 10.3390/ijms232416040 (PMC9781049; doi:10.3390/ijms232416040)
Supplement: Supplementary file 1 [file ijms-23-16040-s001.zip › ijms-2050945-supplementary.pdf]

SUPPLEMENTARY MATERIALS

Influence of metal salts addition on physical and electrochemical properties of ethyl and propylammonium nitrate.

David Ausín, José L. Trenzado, Mireille Turmine, Luis M. Varela, Oscar Cabeza; Elisa González Romero and Luisa Segade*

* luisa.segade@udc.es

Table S1. Experimental densities (ρ) of PIL mixtures, at different temperatures (T), as function of water (w) and the incorporated cation content (ICP-MS and $[Me^{n+}]$).

		ρ (g·cm⁻³)							
	**PLI**	**EAN**						**PAN**	
	**Incorporated Salt**	$LiNO_3$	$LiNO_3$	$LiNO_3$	$LiNO_3$	$Mg(NO_3)_2$	$Al(NO_3)_3$	$LiNO_3$	$Al(NO_3)_3$
	**ICP-MS ($mg \cdot g^{-1}$)**	3.3 ± 0.1	6.5 ± 0.3	8.2 ± 0.4	9.9 ± 0.6	32 ± 1	28 ± 1	8.8 ± 0.1	31 ± 2
	**$[Me^{n+}]$ (m)**	0.49	1.00	1.29	1.59	1.68	1.40	1.40	1.53
	**w (ppm)**	4730 ± 50	3430 ± 50	3270 ± 50	4300 ± 50	19320 ± 60	40050 ± 70	3890 ± 50	1960 ± 50
**T (°C)**	**95**	1.1862	1.2019	1.2059	1.2162	1.2901	1.2824		
	**90**	1.1892	1.2048	1.2070	1.2189	1.2936	1.2852		
	**85**	1.1918	1.2077	1.2092	1.2218	1.2970	1.2885		
	**80**	1.1946	1.2106	1.2133	1.2248	1.3004	1.2919		
	**75**	1.1974	1.2135	1.2162	1.2278	1.3037	1.2951		
	**70**	1.2003	1.2165	1.2192	1.2308	1.3071	1.2985		
	**65**	1.2033	1.2195	1.2222	1.2338	1.3105	1.3020		
	**60**	1.2062	1.2225	1.2252	1.2368	1.3139	1.3055		
	**55**	1.2092	1.2255	1.2284	1.2399	1.3173	1.3090	1.1823	
	**50**	1.2122	1.2285	1.2315	1.2429	1.3208	1.3123	1.1855	
	**45**	1.2153	1.2316	1.2347	1.2460	1.3242	1.3160	1.1883	
	**40**	1.2183	1.2347	1.2379	1.2492	1.3277	1.3197	1.1917	
	**35**	1.2214	1.2378	1.2411	1.2523	1.3312	1.3234	1.1948	
	**30**	1.2245	1.2410	1.2452	1.2555	1.3347	1.3271	1.1981	
	**25**	1.2277	1.2442	1.2483	1.2587	1.3382	1.3308	1.2014	1.2418
	**20**	1.2308	1.2473	1.2516	1.2619	1.3418	1.3346	1.2045	
	**15**	1.2340	1.2506	1.2548	1.2651	1.3455	1.3384	1.2077	
	**10**	1.2372	1.2538	1.2581	1.2683	1.3492	1.3422	1.2110	
	**5**	1.2405	1.2570	1.2614	1.2716	1.3531	1.3461	1.2143	

Table S2. Experimental viscosities (η) of PIL mixtures, at different temperatures (T), as function of water (w) and the incorporated cation content (ICP-MS and [Me^{n+}]).

		η (mPa·s)							
	**PLI**	**EAN**						**PAN**	
	**Sal**	$LiNO_3$	$LiNO_3$	$LiNO_3$	$LiNO_3$	$Mg(NO_3)_2$	$Al(NO_3)_3$	$LiNO_3$	$Al(NO_3)_3$
	**ICP-MS (mg·g^{-1})**	3.3 ± 0.1	6.5 ± 0.3	8.2 ± 0.4	9.9 ± 0.6	32 ± 1	28 ± 1	8.8 ± 0.1	31 ± 2
	**[Me^{n+}] (m)**	0.49	1.00	1.29	1.59	1.68	1.40	1.40	1.53
	**w (ppm)**	4730 ± 50	3430 ± 50	3270 ± 50	4300 ± 50	19320 ± 60	40050 ± 70	3890 ± 50	1960 ± 50
**T (°C)**	**95**	7.99	8.62	9.27	9.33	17.13	11.13		
	**90**	8.77	9.47	10.11	10.26	18.94	12.07		
	**85**	9.59	10.44	11.10	11.31	21.09	13.62		
	**80**	10.55	11.47	12.23	12.50	23.69	15.32		
	**75**	11.65	12.71	13.41	13.88	26.8	17.14		
	**70**	12.92	14.16	14.94	15.47	30.6	19.48		
	**65**	14.42	15.86	16.74	17.36	35.3	22.34		
	**60**	16.17	17.88	18.88	19.61	41.0	25.8		
	**55**	18.25	20.28	21.43	22.29	48.1	29.9	34.0	
	**50**	20.74	23.17	24.51	25.5	57.0	35.1	40.0	
	**45**	23.75	26.7	28.3	29.5	68.5	41.6	47.1	
	**40**	27.4	31.0	32.9	34.4	83.3	49.7	56.7	
	**35**	32.0	36.4	38.7	40.6	102.8	60.2	69.2	
	**30**	37.7	43.2	46.0	48.4	128.9	73.8	85.0	
	**25**	44.9	51.8	55.3	58.5	164.6	91.9	105.9	213.3
	**20**	54.1	63.1	67.5	71.6	214.4	116.2	134.1	
	**15**	66.2	77.9	83.6	89.0	286	149.8	173.5	
	**10**	82.3	97.8	105.3	112.7	390	197.0	229.2	
	**5**	104.0	125.0	135.1	145.4	549	265	310	

Table S3. Experimental electrical conductivities (κ) of PIL mixtures, at different temperatures (T), as function of water (w) and the incorporated cation content (ICP-MS and [Me^{n+}]).

					κ ($mS \cdot cm^{-1}$)									
**PLI**	**Sal**	**ICP-MS ($mg \cdot g^{-1}$)**	**[Me^{n+}] (m)**	**w (ppm)**	**T (°C)**									
					**5**	**15**	**25**	**35**	**45**	**55**	**65**	**75**	**85**	**95**
EAN	$LiNO_3$	3.3 ± 0.1	0.49	4730 ± 50	9.77	14.16	19.40	25.4	32.2	39.0	46.4	54.1	61.9	70.7
EAN	$LiNO_3$	6.5 ± 0.3	1.00	3430 ± 50	8.16	12.13	16.87	22.5	29.1	35.9	42.9	50.4	57.4	66.0
EAN	$LiNO_3$	8.2 ± 0.4	1.29	3270 ± 50	7.69	11.48	16.13	21.6	28.0	34.6	41.7	48.8	56.5	64.6
EAN	$LiNO_3$	9.9 ± 0.6	1.59	4300 ± 50	7.00	10.63	15.14	20.4	27.0	33.7	40.8	48.2	56.4	64.5
EAN	$LiNO_3$	10.8 ± 0.1	1.77	14840 ± 50	6.33	9.75	14.04	19.22	25.4	32.1	39.4	47.2	55.2	64.2
EAN	$LiNO_3$	12.2 ± 0.4	2.01	2800 ± 50	4.89	7.76	11.39	15.87	21.0	27.1	33.4	40.2	46.7	54.5
EAN	$Ca(NO_3)_2$ (liquid)	49 ± 1	1.54	6600 ± 120	1.965	3.66	5.935	8.86	12.53	16.62	21.4	27.3	32.7	39.1
EAN	$Ca(NO_3)_2$ (solid)	49 ± 1	1.54	6600 ± 120	2.37	3.82	5.59	7.75	9.49	13.67	20.5			
EAN	$Mg(NO_3)_2$	32 ± 1	1.68	19320 ± 60	2.28	4.2	6.59	9.79	13.59	18.13	23.4	29.4	35.2	42.1
EAN	$Al(NO_3)_3$	28 ± 1	1.40	38870 ± 70	5.35	8.3	12.075	16.69	21.9	28.3	34.8	41.5	49.1	57.1
PAN	$LiNO_3$	8.8 ± 0.1	1.40	3890 ± 50	2.49	4.37	6.685	9.64	13.14	17.16	21.7	26.7	32.4	37.8
PAN	$Ca(NO_3)_2$	62 ± 1	2.09	4870 ± 50			0.647							
PAN	$Mg(NO_3)_2$	77 ± 1	6.11	11350 ± 70	0.00058	0.00491	0.02525	0.0927	0.265	0.615	1.200	2.10	3.55	5.28
PAN	$Al(NO_3)_3$	31 ± 2	1.53	1960 ± 50	1.961	3.38	5.13	7.31	9.95	13.06	16.54	20.4	24.7	29.6

Table S4. Experimental refractive index (n_D) of PIL mixtures, at 25 °C, as function of water (w) and the incorporated cation content (ICP-MS and [Me^{n+}]).

**25 °C**				
**EAN**				
**Sal**	**ICP-MS (mg·g^{-1})**	**[Me^{n+}] (m)***	**w (ppm)**	**n_D**
$LiNO_3$	3.3 ± 0.1	0.49	4730 ± 50	1.45549
$LiNO_3$	6.5 ± 0.3	1.00	3430 ± 50	1.45677
$LiNO_3$	8.2 ± 0.4	1.29	3270 ± 50	1.45768
$LiNO_3$	9.9 ± 0.6	1.59	4300 ± 50	1.45848
$Mg(NO_3)_2$	32 ± 1	1.68	19320 ± 60	1.46218
$Al(NO_3)_3$	28 ± 1	1.40	40050 ± 70	1.45960
**PAN**				
**Sal**	**ICP-MS (mg·g^{-1})**	**[Me^{n+}] (m)***	**w (ppm)**	**n_D**
**$LiNO_3$**	8.8 ± 0.1	1.40	3890 ± 50	1.45910
**$Al(NO_3)_3$**	31 ± 2	1.53	1960 ± 50	1.46246

Table S5. Experimental surface tension (σ) of PIL mixtures, at 25 °C, as function of water (w) and the incorporated cation content (ICP-MS and [Me^{n+}]).

**25 °C**				
**EAN**				
**Sal**	**ICP-MS (mg·g^{-1})**	**[Me^{n+}] (m)***	**w (ppm)**	**λ (mN·m^{-1})**
**$LiNO_3$**	3.3 ± 0.1	0.49	4730 ± 50	48.15
**$LiNO_3$**	6.5 ± 0.3	1.00	3430 ± 50	48.20
**$LiNO_3$**	8.2 ± 0.4	1.29	3270 ± 50	48.22
**$LiNO_3$**	9.9 ± 0.6	1.59	4300 ± 50	48.70
**$Mg(NO_3)_2$**	32 ± 1	1.68	19320 ± 60	49.65
**$Al(NO_3)_3$**	28 ± 1	1.40	40050 ± 70	45.86
**PAN**				
**Sal**	**ICP-MS (mg·g^{-1})**	**[Me^{n+}] (m)***	**w (ppm)**	**σ (mN·m^{-1})**
**$LiNO_3$**	8.8 ± 0.1	1.40	3890 ± 50	39.06
**$Al(NO_3)_3$**	31 ± 2	1.53	1960 ± 50	37.76

Table S6. Anodic and cathodic limits and electrochemical potential windows (EPW) of PIL mixtures, at 25 °C and $E^{0'}$ (Fc) = 0 V, in each working electrode (WE) and scan rate (ν).-< and > are used when the criteria of 5 mA·cm^{-2} was not reached.

**Sample**	**WE**	**ν (V·s^{-1})**	**Cathodic limit (V)**	**Anodic limit (V)**	**EPW (V)**
**EAN**	DPt	0.100	-0.998	1.732	2.730
**EAN + Li**	DPt	0.100	-1.019	1.761	2.780
**EAN + Ca**	DPt	0.100	-1.074	1.783	2.857
**EAN + Mg**	DPt	0.100	-1.024	1.821	2.845
**EAN + Al**	DPt	0.100	0.429	1.774	1.345
**EAN**	DPt	0.010	-1.028	1.747	2.775
**EAN + Li**	DPt	0.010	-1.059	1.771	2.830
**EAN + Ca**	DPt	0.010	-1.069	1.791	2.860
**EAN + Mg**	DPt	0.010	-1.045	1.806	2.851
**EAN + Al**	DPt	0.010	0.590	1.779	1.189
**EAN**	DPt	0.001	-1.075	1.756	2.831
**EAN + Li**	DPt	0.001	-1.099	1.781	2.880
**EAN + Ca**	DPt	0.001	-1.120	1.811	2.931
**EAN + Mg**	DPt	0.001	<-0.984	1.816	>2.800
**EAN + Al**	DPt	0.001	0.610	1.789	1.179
**EAN**	DGC	0.100	-1.357	1.670	3.027
**EAN + Li**	DGC	0.100	-1.645	1.694	3.339

**EAN + Ca**	DGC	0.100	-1.746	1.734	3.480
**EAN + Mg**	DGC	0.100	-1.560	1.764	3.324
**EAN + Al**	DGC	0.100	0.493	1.732	1.239
**EAN**	DGC	0.010	-1.373	1.729	3.102
**EAN + Li**	DGC	0.010	-1.564	1.754	3.318
**EAN + Ca**	DGC	0.010	-1.610	1.779	3.389
**EAN + Mg**	DGC	0.010	<-1.650	1.850	>3.500
**EAN + Al**	DGC	0.010	0.392	1.772	1.380
**EAN**	DGC	0.001	-1.343	1.780	3.123
**EAN + Li**	DGC	0.001	-1.700	1.805	3.505
**EAN + Ca**	DGC	0.001	-1.690	1.830	3.520
**EAN + Mg**	DGC	0.001	<-1.555	1.855	>3.410
**EAN + Al**	DGC	0.001	0.211	1.873	1.662
**EAN**	SPE	0.100	-2.060	1.900	3.960
**EAN + Li**	SPE	0.100	-1.972	1.826	3.798
**EAN + Ca**	SPE	0.100	<-2.075	>1.929	>4.004
**EAN + Mg**	SPE	0.100	-2.065	1.880	3.945
**EAN + Al**	SPE	0.100	-1.806	1.758	3.564
**PAN**	DPt	0.100	-1.031	1.694	2.725
**PAN + Li**	DPt	0.100	-1.066	1.714	2.780
**PAN + Ca**	DPt	0.100	-1.424	2.095	3.519
**PAN + Mg**	DPt	0.100	<-8.750	>6.071	>14.821
**PAN + Al**	DPt	0.100	-0.559	1.707	2.266
**PAN**	DPt	0.010	-1.071	1.708	2.779
**PAN + Li**	DPt	0.010	-1.091	1.724	2.815
**PAN + Ca**	DPt	0.010	-1.414	2.036	3.450
**PAN + Mg**	DPt	0.010			
**PAN + Al**	DPt	0.010	-1.057	1.728	2.785
**PAN**	DPt	0.001	-1.117	1.734	2.851
**PAN + Li**	DPt	0.001	-1.131	1.749	2.880
**PAN + Ca**	DPt	0.001	-1.253	1.950	3.203
**PAN + Mg**	DPt	0.001			
**PAN + Al**	DPt	0.001	-1.133	1.758	2.891
**PAN**	DGC	0.100	-1.554	1.628	3.182
**PAN + Li**	DGC	0.100	-1.544	1.644	3.188
**PAN + Ca**	DGC	0.100	-1.761	1.749	3.510
**PAN + Mg**	DGC	0.100	<-5.748	>6.255	>12.003
**PAN + Al**	DGC	0.100	-0.251	1.648	1.899
**PAN**	DGC	0.010	-1.463	1.668	3.131
**PAN + Li**	DGC	0.010	-1.564	1.694	3.258
**PAN + Ca**	DGC	0.010	-1.806	1.814	3.620
**PAN + Mg**	DGC	0.010			
**PAN + Al**	DGC	0.010	-1.555	1.748	3.303
**PAN**	DGC	0.001	-1.489	1.845	3.334
**PAN + Li**	DGC	0.001	-1.665	1.910	3.575
**PAN + Ca**	DGC	0.001	-1.867	2.046	3.913
**PAN + Mg**	DGC	0.001			
**PAN + Al**	DGC	0.001	-1.837	1.925	3.762
**PAN**	SPE	0.100	-1.647	1.429	3.076
**PAN + Li**	SPE	0.100	-1.784	1.497	3.281
**PAN + Ca**	SPE	0.100	-1.857	1.605	3.457
**PAN + Mg**	SPE	0.100	<-2.038	>1.966	>4.004
**PAN + Al**	SPE	0.100	-1.550	1.463	3.013

Table S7. Fitting of the calculated data for EAN +$LiNO_3$ as function of the metal cation content: Temperature (T), parameters of equation (4) and coefficient of determination (R^2).

		κ (mS·cm⁻¹)		
**T (°C)**	Q_{IL} (mS·cm^{-1})	A_1 (mS·cm^{-1}·m^{-1})	A_2 (mS·cm^{-1}·m^{-2})	R^2
**5**	10.461	-3.1849	0.35201	0.996
**15**	15.151	-4.5151	0.65851	0.994
**25**	20.635	-5.7264	0.91112	0.997
**35**	26.789	-6.7131	1.1033	0.994
**45**	33.616	-7.4215	1.3529	0.998
**55**	40.846	-8.7695	1.8948	0.994
**65**	48.428	-10.203	2.5120	0.993
**75**	56.444	-11.506	2.9750	0.996
**85**	64.717	-14.095	4.4929	0.998
**95**	73.045	-15.067	5.0842	0.9997
		**η (mPa·s)**		
**T (°C)**	**Q_{IL} (mPa·s)**	**A_1 (mPa·s·m^{-1})**	**A_2 (mPa·s·m^{-2})**	**R^2**
**5**	87.1	49.07	-5.527	0.998
**10**	69.6	37.53	-4.808	0.998
**15**	56.6	28.89	-3.910	0.998
**20**	46.6	22.83	-3.348	0.998
**25**	39.0	17.88	-2.591	0.997
**30**	32.9	14.80	-2.407	0.997
**35**	28.1	12.02	-1.984	0.996
**40**	24.26	98.63	-1.672	0.996
**45**	21.02	83.44	-1.432	0.997
**50**	18.42	70.41	-1.248	0.995
**55**	16.30	5.892	-1.019	0.996
**60**	14.49	5.083	-0.8920	0.995
**65**	12.98	4.370	-0.7829	0.994
**70**	11.67	3.825	-0.6926	0.993
**75**	10.55	3.315	-0.5858	0.992
**80**	9.570	3.000	-0.5530	0.992
**85**	8.739	2.714	-0.5271	0.993
**90**	8.009	2.385	-0.4596	0.989
**95**	7.363	2.066	-0.3727	0.987
		**ρ (g·cm⁻³)**		
**T (°C)**	**Q_{IL}** (g·cm^{-3})	**A_1** (g·cm^{-3}·m^{-1})	**A_2** (g·cm^{-3}·m^{-2})	**R^2**
**5**	1.2227	4.0842	-6.1048	0.999995
**10**	1.2197	4.0271	-5.8155	0.99997
**15**	1.2167	3.9886	-5.6910	0.99994
**20**	1.2138	3.9250	-5.3840	0.99994
**25**	1.2108	3.9266	-5.4412	0.99993
**30**	1.2078	3.8524	-5.0549	0.99990
**35**	1.2048	3.8167	-4.9243	0.99990
**40**	1.2018	3.7982	-4.8906	0.99987
**45**	1.1989	3.8105	-5.0272	0.99990
**50**	1.1959	3.7842	-4.9081	0.99988
**55**	1.1930	3.7608	-4.7829	0.99991
**60**	1.1900	3.7718	-4.8516	0.99987
**65**	1.1870	3.7840	-4.9882	0.99989
**70**	1.1840	3.7778	-4.9600	0.99989
**75**	1.1811	3.8023	-5.1020	0.99993
**80**	1.1782	3.8175	-5.2324	0.99998
**85**	1.1752	3.8498	-5.4599	0.999994

**90**	1.1723	3.9158	-5.8515	0.99998
**95**	1.1693	3.8926	-5.5845	0.999995

		n_D		
**T (°C)**	Q_{IL}	A_1	A_2	R^2
**25**	1.45410	$2.94511 \cdot 10^{-3}$		0.993

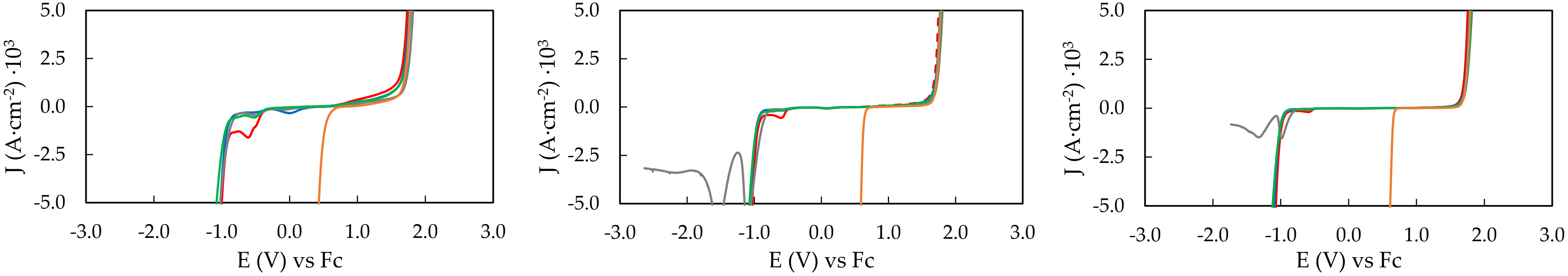

(a) **(b)** **(c)**

Figure S1. Voltamperograms of EAN (▬) and its mixtures with $LiNO_3$ (▬), $Ca(NO_3)_2$ (▬), $Mg(NO_3)_2$ (▬) and $Al(NO_3)_3$ (▬). Voltamperograms obtained with DPt as working electrode at 0.100 (**a**), 0.010 (**b**) and 0.001 (**c**) $V{\cdot}s^{-1}$.

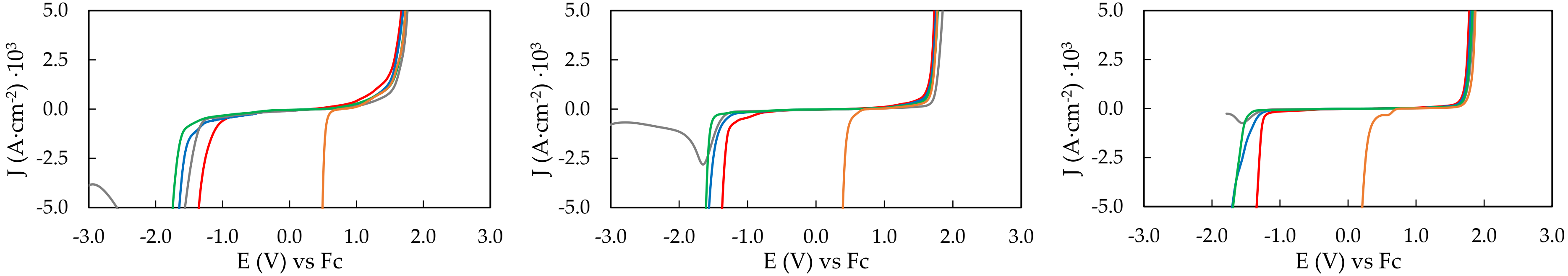

(a) **(b)** **(c)**

Figure S2. Voltamperograms of EAN (▬) and its mixtures with $LiNO_3$ (▬), $Ca(NO_3)_2$ (▬), $Mg(NO_3)_2$ (▬) and $Al(NO_3)_3$ (▬). Voltamperograms obtained with DGC as working electrode at 0.100 (**a**), 0.010 (**b**) and 0.001 (**c**) $V{\cdot}s^{-1}$.

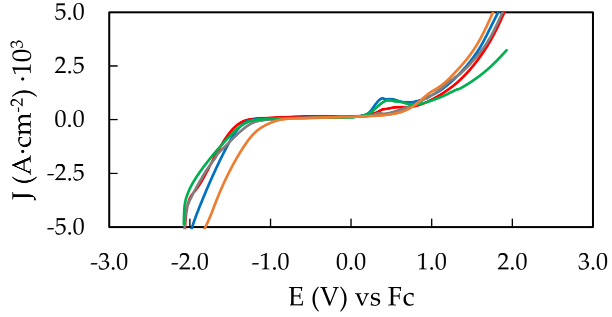

Figure S3. Voltamperograms of EAN (▬) and its mixtures with $LiNO_3$ (▬), $Ca(NO_3)_2$ (▬), $Mg(NO_3)_2$ (▬) and $Al(NO_3)_3$ (▬). Voltamperograms obtained with SPE as working electrode at 0.100 $V{\cdot}s^{-1}$.

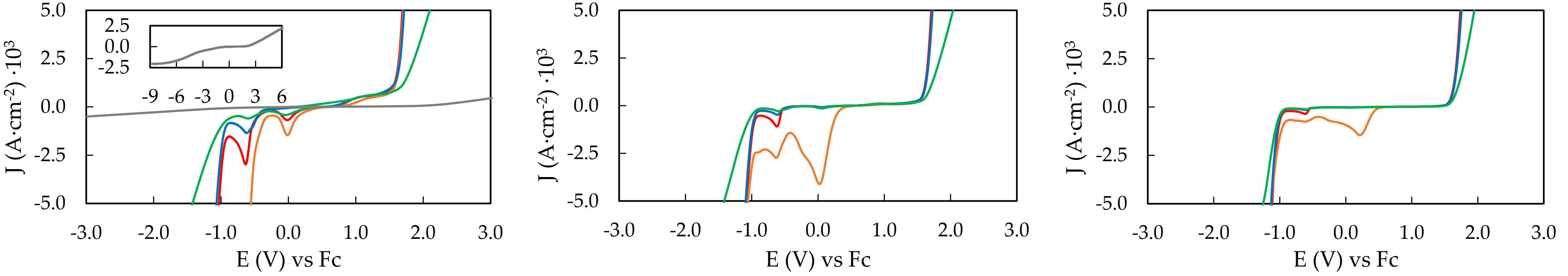

(a) (b) (c)

Figure S4. Voltamperograms of PAN (▬) and its mixtures with $LiNO_3$ (▬), $Ca(NO_3)_2$ (▬), $Mg(NO_3)_2$ (▬) and $Al(NO_3)_3$ (▬). Voltamperograms obtained with DPt as working electrode at 0.100 (**a**), 0.010 (**b**) and 0.001 (**c**) $V{\cdot}s^{-1}$.

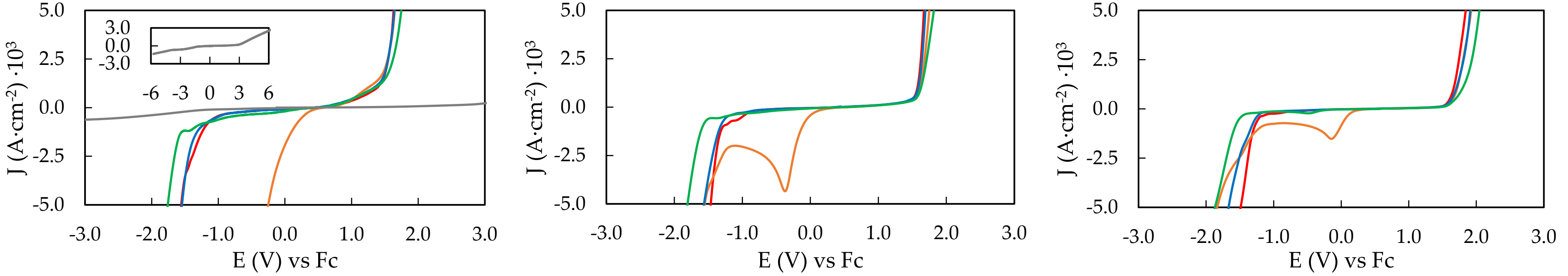

(a) (b) (c)

Figure S5. Voltamperograms of PAN (▬) and its mixtures with $LiNO_3$ (▬), $Ca(NO_3)_2$ (▬), $Mg(NO_3)_2$ (▬) and $Al(NO_3)_3$ (▬). Voltamperograms obtained with DGC as working electrode at 0.100 (**a**), 0.010 (**b**) and 0.001 (**c**) $V{\cdot}s^{-1}$.

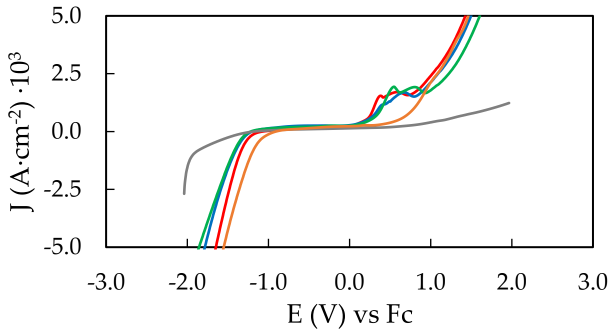

Figure S6. Voltamperograms of PAN (▬) and its mixtures with $LiNO_3$ (▬), $Ca(NO_3)_2$ (▬), $Mg(NO_3)_2$ (▬) and $Al(NO_3)_3$ (▬). Voltamperograms obtained with SPE as working electrode at 0.100 $V{\cdot}s^{-1}$.
